# ChillsDB: A Gold Standard for Aesthetic Chills Stimuli

**DOI:** 10.1038/s41597-023-02064-8

**Published:** 2023-05-20

**Authors:** Felix Schoeller, Abhinandan Jain, Adam Haar Horowitz, Grace Yan, Xiaoxiao Hu, Pattie Maes, Roy Salomon

**Affiliations:** 1 Massachusetts Institute of Technology, Media Lab, Cambridge, USA; 2 Gonda Multidisciplinary Brain Centre, Bar Ilan University, Ramat Gan, Israel; 3 Institute for Advanced Consciousness Studies, Santa Monica, Califronia USA; 4 Wellesley College, Wellesley, USA

**Keywords:** Physiology, Psychology, Social sciences

## Abstract

We introduce ChillsDB the first validated database of audiovisual stimuli eliciting aesthetic chills (goosebumps, psychogenic shivers) in a US population. To discover chills stimuli “in the wild”, we devised a bottom-up, ecologically-valid method consisting in searching for mentions of the emotion’ somatic markers in user comments throughout social media platforms (YouTube and Reddit). We successfully captured 204 chills-eliciting videos of three categories: music, film, and speech. We then tested the top 50 videos in the database on 600+ participants and validated a gold standard of 10 stimuli with a 0.9 probability of generating chills. All ChillsDB tools and data are fully available on GitHub for researchers to be able to contribute and perform further analysis.

## Background & Summary

Aesthetic chills are a universal marker of human peak experiences across the arts, sciences and world religions^1–3^. Best characterized as the feeling of cold down the spine or goosebumps while engaging with music or film, chills are the sensation associated to shivers: short thermogenic tremors of skeletal muscles. Though ordinarily involved in the regulation of temperature (or as an immune response during fever), chills can also be triggered by information-related processes (e.g., music, stories, speeches), independently of changes in temperature (i.e., psychogenic shivers, thereafter “chills”).

As a conscious, measurable, and universal emotion, with clear delineated correlates and a wide range of behavioural consequences, chills are a promising field of study for affective and social neuroscience^4,5^. However, no validated database of stimuli exists to elicit aesthetic chills. In psychology and neuroscience, using audiovisual stimuli is a standard procedure for generating and studying emotional reactions, both in humans and primates. Emotional stimulation includes images^6^, audio^7^, video^8–13^, or text^14^ and provides a reliable way to elicit emotion in controlled laboratory settings.

Chills seem to be a universal emotional phenomenon found across human culture and languages^2^. As a predictor for the personality trait Openness to Experience^2^, chills can be generated from a wide range of media: music, films, paintings, poetry, science, mathematics, religion, rituals^1^. Studies report a number of behavioural effects such as increased altruism^15^, pleasure^1^ and reward^1^. More recently, chills have been related to learning and meaning-making^1^, and demonstrated effects on cognitive functions such as attention and memory^16^. As an emotional peak, chills also have downstream effects on valence and arousal^17^, and physiologic factors such as heart rate^18^, pupil dilatation^19^, skin conductance^19^, and muscle contractions^20^.

However, most research focuses solely on music as a stimulus^15,21,22^, and researchers usually ask participants to bring their own chills-eliciting music to the laboratory^15^. Some databases exist for emotional labels that include chills as a signature (e.g., awe, being moved, kama muta), but none strictly focused on chills. To fill this gap, we introduce ChillsDB, a validated database of chill-eliciting stimuli for a US population. The database consists of 204 chill eliciting audiovisual stimuli collected from social media platforms (YouTube and Reddit) by counting mentions of keywords of somatic markers of aesthetic chills in the user comments. In a subsequent study, we validated 50 stimuli videos (t < 10 minutes duration) in their propensity to elicit chills. The goal was to capture 10 videos with a probability to generate chills higher than 0.7 (i.e., 7 participants out of 10 should experience them when exposed to the stimulus).

## Methods

### Database design

We used online social media platforms, YouTube and Reddit, as sources to gather stimulus which elicits chills. For data collection, we developed a Python-based tool to find stimuli distributed across social media platform using breadth-first search algorithm^23^. The tool uses a seed video as a starting point to search for related videos using the social media platforms’s recommendation system. For determining if the video qualifies as stimulus, the tool uses a predefined dictionary to find word occurrences in the user comments of the video. The tool then traverses in the network of the seed video and checks if the video qualifies as a stimulus. If the video qualifies, the tool traverses in the network of the qualified video to find more videos and repeats the process. Else if the video does not qualify, the algorithm continues searching in the network of the seed video.

For the video to qualify as a chills eliciting stimulus, we use mentions of somatic markers of the shiver in user comments as a way to determine the propensity of video to elicit chills. Based on the existing literature, we used a somatic marker dictionary containing: ‘frisson’, ‘chill’, ‘goosebump’, ‘gooseflesh’ as the keywords for somatic reference to define aesthetic chills. Based on the number of cumulative occurrences of the keyword in the user comment section, the stimulus was selected to be part of the database. For the video to be selected as stimulus, we used the criterion of having at least 10 occurrences of the somatic marker dictionary in the stimulus comments, with a total amount of comments greater than 100.

All the qualified videos were saved in a Comma Separated Values (.csv) file and videos with high mentions of somatic markers were reused as seed videos to find newer videos. We collected the total comments, likes count, dislike count and somatic marker occurrences from the qualified video. We used a similar process to gather stimulus from the Reddit platform, searching for the dictionnary in the Reddit comments. We found stimulus in Reddit from forums ‘r/Frisson’, ‘r/inspiring’ and ‘r/inspiration’. Since Reddit posts had significantly lower comment counts than YouTube, we did not use minimum somatic marker occurrence criteria to select the stimulus. From YouTube and Reddit we collected 204 stimulus samples (100 in YouTube and 104 in Reddit). From the 204 stimulus samples, we selected the top 50 stimulus that mentioned the dictionary keywords with the highest frequency, to evaluate their propensity in generating chills.

To validate the database stimulus, we used the Prolific online platform to recruit participants. We also set up an online website to present the stimuli and collect user data. Participants from Prolific were redirected to the online platform and completed the survey after watching the stimulus. Our online platform comprised two attention checks^24^, one before and one after the study so as to ensure high quality data.

### Participants

660 subjects participated in our experiment (Mean age = 33.6, 50% males, 49.5% female and 0.5% = other). We removed 100 participants who reported an aberrant proportion of chills as compared to the rest of the sample (N > 10) and did not fulfil the two attention checks. Participants were recruited on an online platform and were screened for psychiatric conditions or neurologic disorders. All the participants reside in the United States of America and practised English as their first language. 75% White, 7.9% Multiracial, 8.4% Asian, 3.6% African American, 3.9% Hispanic, and 2% Middle Eastern. An additional sample of 130 participants (Mean age = 37, 60 males) rated the top 6 stimuli, we removed 4 participants who did not fulfil the attention check. The participants were paid proportional to their time in the study at approx hourly rate of USD 11.79.

### Validation

To validate the gold standard, we conducted an online study on Prolific crowdsourcing platform to evaluate the emotional effect of chill-inducing videos. Participants were first screened for neurologic disorders and randomly assigned to one of ChillsDB top 50 videos. Before the video, they were given a definition of “the feeling of emotional chills and shivers” as “the feeling of cold down your spine that are NOT related to temperature or sickness but that are caused by some strong emotions”. Participants were asked to report their age, gender, ethnicity, frequency of daily chills (1 - never; 5 - always), NEO item 188 score (“Sometimes when I am reading poetry or looking at a work of art, I feel a chill or wave of excitement”) (1 - Strongly Disagree; 5- Strongly Agree). Participants were then exposed to the audiovisual stimulus from the database and asked to report whenever they experienced chills by pressing a large button on the right side of the screen. Only once the stimulus is over are they allowed to continue to the next part of the study. After the exposure they were asked to report the intensity of the chills, and rate their experience in terms of valence and arousal all on a 5 point Likert scale.

To ensure the quality of data we implemented two attention checks, one before the stimulus and one after, to determine if participants were paying attention to questions (Oppenheimer *et al*., 2009). The attention checks constituted selecting the correct response to a described question, e.g. “Please select ‘strongly agree’ for this question”. Participants were compensated based on time spent in the study at an approximately hourly rate of USD 11.72. Each experiment lasted approximately 10 minutes and was well-received by the participant. Some of the participants even wrote to the authors a personal email to thank them for the experience (e.g., “I just wanted to take a moment and tell you that I thoroughly enjoyed this study and found it also to be a unique experiment”).

### Ethics

The experiment is in compliance with the Helsinki Declaration. The study was approved by the Committee on the Use of Humans as Experimental Subjects at MIT. All participants gave their voluntary informed consent and we followed the Ethics Code of the American Psychological Association. All participants were informed about the purpose of the research, their right to decline to participate and to withdraw from the experiment, and the limits of confidentiality. We also provided them with a contact for any questions concerning the research and with the opportunity to ask any questions regarding the phenomenon under study (aesthetic chills) and receive appropriate answers. All participants reacted positively to the experiment and were thankful for the opportunity to learn about the phenomenon.

## Data Records

ChillsDB consists in 5 .csv sheets available under a CC0 1.0 license on the associated Harvard Dataverse^25^. A simple user interfaces allows anyone to see the YouTube videos (www.chillsdb.com). One can also explore the database based on 1) the platform parsed (YouTube or Reddit) these sheets include the comments from the users, 2) the validated stimuli (top 100, top 50, top 10 have 10 participants per videos and the top 6 has 30 participants per videos), 3) the type of stimuli (music, film, or speech), and finally 4) the participant data (with 2 samples of participants, the first 600 sample tested on the top 50, and the second 130 sample tested on the top 6).

1. YouTube: 93 English videos captured during the YouTube breadth-first search. This file includes YouTube Video ID, Title of the video, Total number of comments, Total number of Likes, Total Number of Dislikes, the results from the sentiment analysis (Positive, Neutral, Stimulating), the count of each dictionary terms (Frisson, chill, gooseflesh, goosebumps) and the comments from users.
2. Reddit: 104 videos captured during the Reddit search. This file includes YouTube Video ID, the SubReddit where it was found, the title of the video, the number of upvotes and downvotes, the ID of the video, the URL, the total number of comments and the comments from users.
3. Top 100: videos from the YouTube and Reddit batch with the most chills comments as a function of total number of comments. The criteria used for YouTube were the highest mentions of the somatic dictionary. We chose the top 15 Reddit videos and images with the highest amount of upvotes and which were not in the YouTube results.
4. Top 10: videos with the highest chills ratio (i.e., the probability of chills as calculated by the number of chills occurrence divided by number of participants) validated on 10 participants each.
5. Top 6: Chills gold standard of validated videos with the highest chills ratio (i.e., the probability of chills as calculated by the number of chills occurrence divided by number of participants) validated on 30 participants each.

### Stimuli

A simple website was installed to access the stimuli (www.chillsdb.com). As mentioned in the previous subsection, the stimuli can be explored together with their associated data in the corresponding sheet based on 1) stimuli type (music, film, or speech) or 2) their platform of origin. For each stimulus, the timing of when the chills is most likely to occur is also included. To access the stimuli from the database, enter the listed video ID after the following:

https://www.youtube.com/watch?v=

Due to copyright reasons, all stimuli were stored on a private server. Please contact the authors to be granted access to the .mp4 of the stimuli.

## Technical Validation

Out of the 50 selected videos, we found 4 types of videos: film, music, speech, and dance. Each video had on average 11.2 participants (min 8, max 24). Given the disparity in group sizes for each video, we calculated the ratio of chills report divided by the number of participants for each video (Chills Ratio). The average Chills Ratio in our sample is 0.7 (Max = 1, Min = 0.3). Each video has on average 2.5 chills (max = 4.2, min = 0.4).

**Fig. 1:**
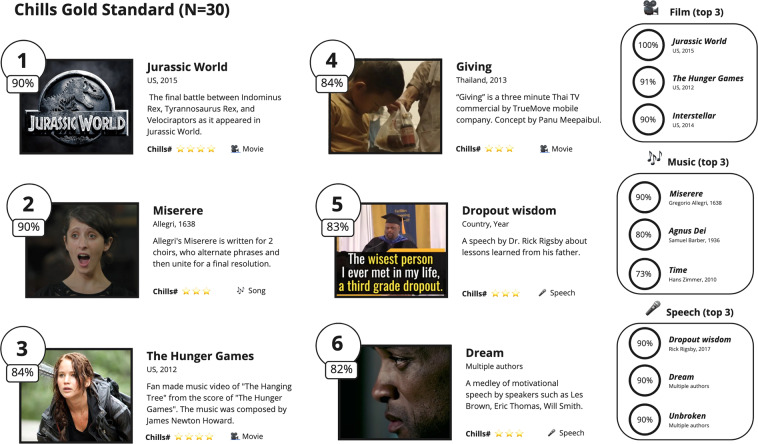
A gold standard for aesthetic chills: the top 6 validated videos from our study (N = 30), including top 3 for each categories (film, music, and speech). All stimuli have a probability ≥0.8 to elicit chills in a US population.

We then proceed to evaluate the top 10 videos of the batch (see Fig. 1). The top 10 videos have a probability greater than or equal to 0.9), which generate on average 2.3 chills. Hence, the amount of chills remains the same even in top videos. These videos have on average 11 participants, 10 of whom reported chills. Compared to the videos of the Zickfeld *et al*. study (M = 0.77, SD = 0.12, our chills ratio is significantly higher (M = 0.91, SD = 0.03).

We then tested the top 6 videos (top 2 films, top 2 music, top 2 speech) on an additional sample of 130 participants, bringing the total of participants per videos to N = 30. The results confirmed the prior analysis with all videos eliciting chills in more than 80% of the participants with two videos (“Jurassic Park” and “Miserere”) eliciting chills in 90% of the participants.

## Usage Notes

We introduce ChillsDB, the first validated dataset for research on aesthetic chills. It includes a mix of films, music, and speeches, which generate chills in a US population (Fig. 1). We conclude that the technique employed here functions well and calls for further research with other somatic markers (e.g., tears for sadness, gasping for awe, nausea for disgust). All the resources employed in this article are fully accessible on GitHub and Harvard Dataverse.

We envision that ChillsDB will allow for at least 3 types of future research otherwise impossible:

- ChillsDB videos can be used to map susceptibility of various psychiatric disorders to reward and allow “stress” test for diagnosing various mood disorders in the form described in^26^.
- ChillsDB can be used as material for affective neuroscience research to drive affective response in a controlled reliable way. Furthermore, it provides a rich set of stimuli for emotion-centered psychotherapy, to improve emotional regulation and interoceptive awareness in Emotion Focused Therapy.
- Finally, ChillsDB also offers valuable materials for intercultural studies in the social and human sciences to study psychosocial similarities and differences in terms of music, speech, and films in fields such as musicology, aesthetics, linguistics, and narratology.

Future research should enrich the database with new content, further classify and analyse the dataset, and test the gold standard with new populations (Middle-East, South America, Asia, Europe, and Africa). Indeed, a limitation of the present study is the fact that we had to rely on subjective reports of chills due to the online nature of the experiment. Future studies should therefore include physiological measures (e.g. pupil size, GSR, HR, etc.) to validate the degree to which chilling experiences are actually induced by these stimuli and confirm the present findings.

Chills is a promising research field. Several hypotheses have been suggested to account for chills but they remain a challenge to operationalize. Schoeller and Perlovsky have suggested that chills relate to learning rate and that they correspond to a satiation of a vital need for information or knowledge^1,27^. Sarasso and colleagues went further to suggest that they may inhibit motor action for the purpose of knowledge-acquisition^28^. With the exception of NEO PI-R and despite a promising biomarker for peak experiences, chills are still largely exempt from psychometric questionnaires^29^. We hope that this validated gold standard will help advance this promising research area.

## Data Availability

The code for parsing YouTube and Reddit networks is available under an MIT license at https://github.com/ChillsTV/AffectiveStimuliScraper.
